# P-selectin overexpression impairs hematopoietic stem cell homeostasis via inflammatory receptor-mediated proliferation and differentiation

**DOI:** 10.1038/s41419-025-08050-9

**Published:** 2025-10-21

**Authors:** Wei He, Huandi Qiu, Yunyu Feng, Qiang Qiu, Li Zheng, Cong Pan, Xue Cui, Yuanyuan Sun, Bochuan Wang, Yiguo Hu

**Affiliations:** 1 State Key Laboratory of Biotherapy, West China Hospital, Sichuan University, Chengdu, China; 2 Department of Clinical Translational Innovation Center and Molecular Medicine Research Center, West China Hospital, Sichuan University, Chengdu, China; 3 Department of Hematology and Research Laboratory of Hematology, West China Hospital, Sichuan University, Chengdu, China; 4 School of Biological Sciences, Guizhou Education University, Guiyang, China

**Keywords:** Haematopoietic stem cells, Chronic lymphocytic leukaemia, Senescence, Chronic inflammation

## Abstract

Hematopoietic stem cells (HSC) sustain lifelong blood and immune system homeostasis. This study identifies P-selectin as a pivotal regulator of HSC function under aging and inflammatory stress. We observed pronounced *Selp* upregulation in aged HSC and inflammatory contexts, which drives excessive proliferation and differentiation while depleting their long-term self-renewal capacity. Using tissue-specific *Selp* overexpression models, we demonstrate that chronic *Selp* elevation disrupts HSC polarity, promotes oxidative stress accumulation, and induces genomic instability. Over time, sustained *Selp* expression leading to LT-HSC exhaustion and impaired hematopoietic reconstitution. Single-cell transcriptomics revealed that *Selp* enforces a pro-inflammatory transcriptional program in HSC, hyperactivating IFN-γ and PI3K-AKT-MOTR signaling pathways. Mechanistically, P-selectin directly interacted with IFNγR1 on the HSC surface, which driving activation of JAK1-STAT1 and PI3K-AKT-mTOR signaling axes. Notably, *Selp* overexpression suppresses the pathogenic capacity of leukemia stem cells (LSC), highlighting potential therapeutic implications. Our findings established P-selectin as a molecular nexus linking chronic inflammation and aging to hematopoietic decline, with dual therapeutic implications: targeting P-selectin may mitigate age-related hematopoietic dysfunction while offering a strategy to selectively impair LSC activity in malignancies.

## Highlights

- *Selp* is significantly elevated in aged HSC and under inflammatory conditions, driving HSC into active proliferation and differentiation while diminishing their ability to maintain quiescence.
- *Selp* upregulation disrupts HSC polarity, increases oxidative stress, and promotes genomic instability, long-term *Selp* overexpression leads to HSC exhaustion and reduced hematopoietic reconstitution capacity.
- *Selp* overexpression exacerbates inflammatory signaling in HSC, particularly through IFN-γ-PI3K-AKT-MOTR pathways.
- *Selp* overexpression reduces the pathogenic capacity of leukemia stem cells.

## Introduction

Hematopoietic stem cells (HSC), which occupy the apex of the hematopoietic hierarchy, serve as the primary source of functional blood cells responsible for maintaining systemic homeostasis [1]. Residing predominantly within the adult bone marrow niche, HSC are essential for preserving hematopoietic equilibrium through tightly regulated self-renewal and differentiation [2, 3]. Under steady-state conditions, most HSC maintain quiescence, with only a minor subset undergoing periodic activation to replenish blood lineages and meet basal physiological demands [4]. During inflammatory or infectious insults, HSC are dynamically activated to amplify the production of lineage-committed cells, thereby fulfilling the body’s escalated demand for immune effectors and tissue repair mediators [5]. The functional integrity of HSC is orchestrated by an intricate regulatory network encompassing cytokines, signaling molecules, transcriptional regulators and cell cycle regulators [6–10]. While significant advances have been made in delineating HSC biology, the precise molecular determinants governing HSC behavior—particularly under pathophysiological conditions such as aging, inflammation, and infection—remain incompletely resolved.

Aging is associated with systemic chronic inflammation which drives cellular senescence and tissue dysfunction through mechanisms such as DNA damage, mitochondrial stress, and oxidative imbalance [11]. Factors secreted by senescent cells, collectively termed the senescence-associated secretory phenotype (SASP), perpetuate chronic inflammation and further promote cellular senescence [12]. To explain aging from the perspective of harmful inflammation and weakened immunity, inflammaging was introduced as an evolutionary perspective on immunosenescence, referring to the phenomenon of low-grade, chronic damage resulting from increased inflammation levels within the body [13]. In HSC, inflammageing manifests as skewed differentiation, reduced regenerative capacity, and transcriptional shifts marked by upregulated stress-response genes and downregulated chromatin remodelers [14].

P-selectin (*Selp*), a key mediator of leukocyte-endothelial adhesion during inflammation responses, is transcriptionally and translationally upregulated in endothelial cells and granulocytes under infectious stress [15]. Intriguingly, similar *Selp* elevation is observed in aged HSC, suggesting its role as a shared marker of inflammatory and aging-associated hematopoietic impairment [16]. Inflammatory cytokines like TNF-α and IL-1β drive *Selp* expression via NF-κB activation, while MAPK signaling (particularly p38) and oxidative stress further amplify its surface presentation [17–19]. These mechanisms enhance endothelial cell adhesion functions, facilitating leukocyte recruitment, rolling, adhesion, and migration, thereby intensifying local inflammatory responses. While *Selp*’s role in endothelial-leukocyte interactions is well-established, its HSC-intrinsic functions remain enigmatic.

In this study, we demonstrated that P-selectin was a central molecular mediator governing HSC dysfunction during aging and inflammatory challenge. Tissue-specific *Selp* overexpression induced progressive HSC exhaustion through multifaceted mechanisms including disruption of cellular polarity, accumulation of oxidative stress and elevated genomic instability. Eventually, these effects drove functional collapse of LT-HSC, result in defective multi-lineage reconstitution in competitive transplantation assays. Single-cell RNA sequencing uncovered *Selp*-mediated transcriptional rewiring toward a pro-inflammatory state, characterized by hyperactivation of IFN-γ signaling cascades. Mechanistic studies revealed that *Selp* physically co-localized with IFNγR1, facilitating its membrane clustering and amplifying JAK-STAT signaling, which activates PI3K-AKT-MTOR pathway accelerates the HSC cycle. This mechanism is not only a rapid response module for stress hematopoiesis, but also a key factor in chronic inflammation-induced aging of HSC.

## Results

### Transcription level of *Selp* is elevated in aged HSC

**Fig. 1:**
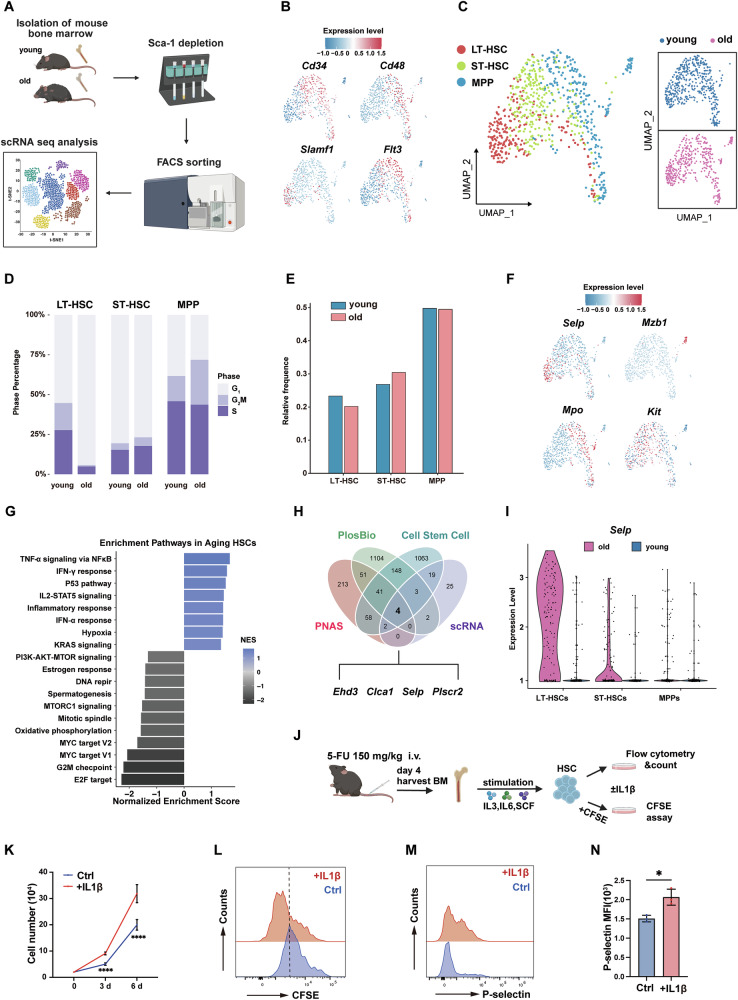
Aged HSC exhibit elevated transcription levels of *Selp.* **A** Schematic diagram of scRNA-seq of HSC from young and old mice. **B** Dot plots showing specific marker genes used for clustering HSC. **C** Left: Cell clusters in isolated HSC were visualized using UMAP. Colors indicate cell types. Each dot represents one cell. Right: Cell clusters in HSC from young and old mice. **D** Cell cycle analysis of LT-HSC, ST-HSC and MPP from young and old mice. **E** Bar plots show the percentage of LT-HSC, ST-HSC and MPP from young and old mice. **F** UMAP plots visualization the expression of four significantly upregulated genes in HSC of aged mice compared to those in young mice. **G** GSEA showcased the significantly upregulated and downregulated signaling pathways in HSC of aged mice compared to those in young mice. **H** Venn diagram displaying 4 candidate genes (*Ehd3, Clca1, Selp, Plscr2*) obtained by taking intersections with genes that are significantly overexpressed in senescent HSC in published articles. **I** *Selp* transcription level in LT-HSC, ST-HSC, MPP from young and old mice. **J** Schematic overview of in vitro IL-1β stimuli assay. **K** Cell expansion of each group in liquid culture (*n* = 3 per group). **L** CFSE dilution assay after 3 days. **M** FACS analysis of P-selectin expression in day 6. **N** Statistic analysis of P-selectin MFI (*n* = 3 per group). Error bars denote mean ± SEM, *P < 0.05, ****P < 0.0001 (*t*-test).

With aging, HSC function progressively deteriorates, accompanied by a decline in both blood cell activity and immune system function [1, 20, 21]. The molecular events driving HSC aging remain incompletely understood. To systematically characterize the dynamic changes of the gene transcriptional differences in HSC during aging, HSC from young (2 months) and aged mice (24 months) were isolated by FACS for single-cell sequencing analysis (Fig. 1A). Based on specific gene expression patterns (Fig. 1B), the cell clusters were manually annotated into three distinct cell types: LT-HSC, ST-HSC and MPP (Fig. 1C). Our data revealed an increased proportion of LT-HSC in the G1 phase in aged mice (Fig. 1D). Combined with the decrease in LT-HSC subset proportions (Fig. 1E), these results suggest that aged LT-HSC are more actively engaged in the cell cycle, with a diminished capacity to maintain quiescence compared to younger mice. Single-cell analysis revealed distinct gene expression patterns in aged mouse HSC. For example, *Selp* was predominantly highly expressed in LT-HSC, while *Mpo* was mainly expressed in MPP (Fig. 1F). Notably, aged HSC exhibited substantial upregulation of several pathways, including TNF-α signaling, IFN-γ response, IFN-α response, IL-2-STAT5 signaling and inflammatory response. In contrast, pathways related to DNA repair (with the exception of p53 signaling), G2M checkpoint and E2F targets exhibited marked downregulation (Fig. 1G). By intersecting the genes upregulated in aged LT-HSC from four previously published datasets, only four genes appear simultaneously in these datasets, *Ehd3*, *Clca1*, *Selp*, and *Plscr2* (Fig. 1H). *Selp* was prioritized for further in-depth investigation due to its established role in HSC adhesion, inflammation, and aging-related pathologies. Further analysis confirmed that while elevated across old HSC subsets, *Selp* expression was most pronounced in LT-HSC (Fig. 1I). Given *Selp’s* role as an inflammatory response gene and the association of aging with chronic inflammation, we investigated whether *Selp* transcription links to inflammatory signaling during aging (Fig. 1J–N). To this end, HSC isolated from young mice were treated with IL-1β, a key aging-associated inflammatory cytokine [22]. Notably, IL-1β treatment accelerated HSC expansion (Fig. 1K), corresponding with increased proliferation rates measured by CFSE assay (Fig. 1L). Critically, P-selectin protein expression was significantly upregulated in IL-1β-treated HSC (Fig. 1M, N). Collectively, aged HSC exhibit markedly elevated *Selp* expression concurrent with broad activation of inflammatory response genes.

### P-selectin is increased in HSC under inflammatory conditions

**Fig. 2:**
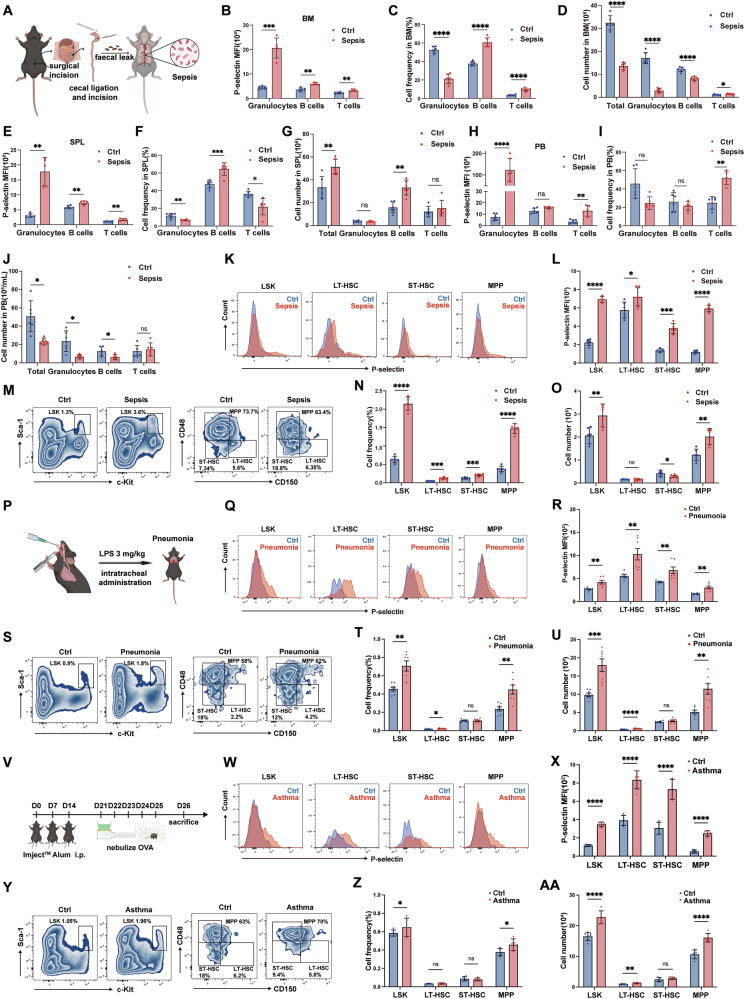
P-selectin is increased in HSC under inflammatory conditions. **A** Schematic of sepsis modeling. **B** Flow cytometry was performed to analyze P-selectin expression levels in bone marrow from sham control and sepsis group mice. The mean fluorescence intensity (MFI) was quantified (*n* = 6 per group). **C**, **D** The percentage and number of granulocytes, B and T cells in BM from sepsis and Ctrl group (*n* = 6 per group). **E** P-selectin expression levels analysis in spleen from sham control and sepsis group mice. The mean fluorescence intensity (MFI) was quantified (*n* = 6 per group). **F**, **G** The percentage and number of granulocytes, B and T cells in SPL from sepsis and Ctrl group (*n* = 6 per group). **H** P-selectin expression levels analysis in PB from sham control and sepsis group mice. The mean fluorescence intensity (MFI) was quantified (*n* = 6 per group). **I**, **J** The percentage and number of granulocytes, B and T cells in PB from sepsis and Ctrl group (*n* = 6 per group). **K** Representative flow cytometry plots showing P-selectin expression levels on HSC from sham surgery control and sepsis group mice. **L** P-selectin MFI of HSCs from sham surgery control and sepsis group mice was then quantified (*n* = 6 per group). **M** Representative flow cytometry plots showing the proportions of LSK, and LT-HSC, ST-HSC, MPP from sepsis model and control group mice. **N** Statistical analysis of the cell proportions shown in (**M**) (*n* = 6 per group). **O** Absolute numbers of LSK, LT-HSC, ST-HSC, MPP from sepsis model and control group mice, calculated based on cell counts (2 femurs + 2 tibias) combined with flow cytometry results (*n* = 6 per group). **P** Schematic of pneumonia modeling. **Q** Representative flow cytometry plots showing P-selectin expression levels on HSC from sham surgery control and pneumonia group mice. **R** P-selectin MFI of HSCs from sham surgery control and pneumonia group mice was then quantified (*n* = 7 per group). **S** Representative flow cytometry plots showing the proportions of LSK, and LT-HSC, ST-HSC, MPP from pneumonia model and control group mice. **T** Statistical analysis of the cell proportions shown in (**S**) (*n* = 7 per group). **U** Absolute numbers of LSK, LT-HSC, ST-HSC, MPP from pneumonia model and control group mice, calculated based on cell counts (2 femurs +2 tibias) combined with flow cytometry results (*n* = 7 per group). **V** Schematic of pneumonia modeling. **W** Representative flow cytometry plots showing P-selectin expression levels on HSC from control and asthma group mice. **X** P-selectin MFI of HSCs from sham surgery control and pneumonia group mice was then quantified (*n* = 5 per group). **Y** Representative flow cytometry plots showing the proportions of LSK, and LT-HSC, ST-HSC, MPP from pneumonia model and control group mice. **Z** Statistical analysis of the cell proportions shown in (**Y**) (*n* = 5 per group). **AA** Absolute numbers of LSK, LT-HSC, ST-HSC, MPP from asthma model and control group mice, calculated based on cell counts (2 femurs +2 tibias) combined with flow cytometry results (*n* = 5 per group). Error bars denote mean ± SEM, ns no significance, *P < 0.05, **P < 0.01, ***P < 0.001, ****P < 0.0001 (*t*-test).

To determine the ubiquity of P-selectin upregulation in HSC during inflammation, we profiled P-selectin expression across multiple experimental inflammatory models. An acute sepsis model (Fig. 2A) revealed significant elevation of P-selectin levels in granulocytes and B/T lymphocytes within bone marrow (BM) compared to sham surgery controls (Fig. 2B). Granulocytes exhibited reduced proportions and absolute counts in BM, while lymphocytes populations showed increased proportions and decreased absolute counts (Fig. 2C, D). Mirroring the BM phenotype, both spleen (SPL) and peripheral blood (PB) exhibited parallel elevations in P-selectin expression alongside similar shifts in immune cell proportions and absolute counts (Fig. 2E–J). Comprehensive profiling of HSC demonstrated consistent P-selectin upregulation across all analyzed subsets, including LT-HSC, ST-HSC, MPP (Fig. 2K, L). Moreover, significant expansion of HSC pools (LT-HSC, ST-HSC, MPP) in BM was observed (Fig. 2M-O). Paralleled the HSC phenotype in sepsis model, the pneumonia model (Fig. 2P) revealed significant P-selectin upregulation in HSC polls (Fig. 2Q, R), concomitant with proportional and numerical expansion of HSC versus controls (Fig. 2S, U). The asthma models (Fig. 2V) exhibited similar HSC phenotypes again (Fig. 2W–AA), mirroring P-selectin upregulation and concomitant expansion of HSC proportions and counts observed in both sepsis and pneumonia models. Together, both models exhibited increased HSC proportions/numbers with amplified P-selectin expression in HSC, while GMP expansion and elevated P-selectin levels were observed in downstream myeloid progenitors (Supplementary Fig. 1A–F). These multi-model analyses establish three hallmark inflammatory features: 1) coordinated expansion of primitive HSC pools, 2) myeloid progenitor amplification, and 3) pan-hematopoietic P-selectin elevation. The consistent temporal coupling between HSC numerical expansion and P-selectin upregulation suggests potential functional interplay, though the causal relationship between P-selectin dynamics and inflammatory hematopoiesis remains undefined.

### *Selp* overexpression causes a hematopoiesis disorder

**Fig. 3:**
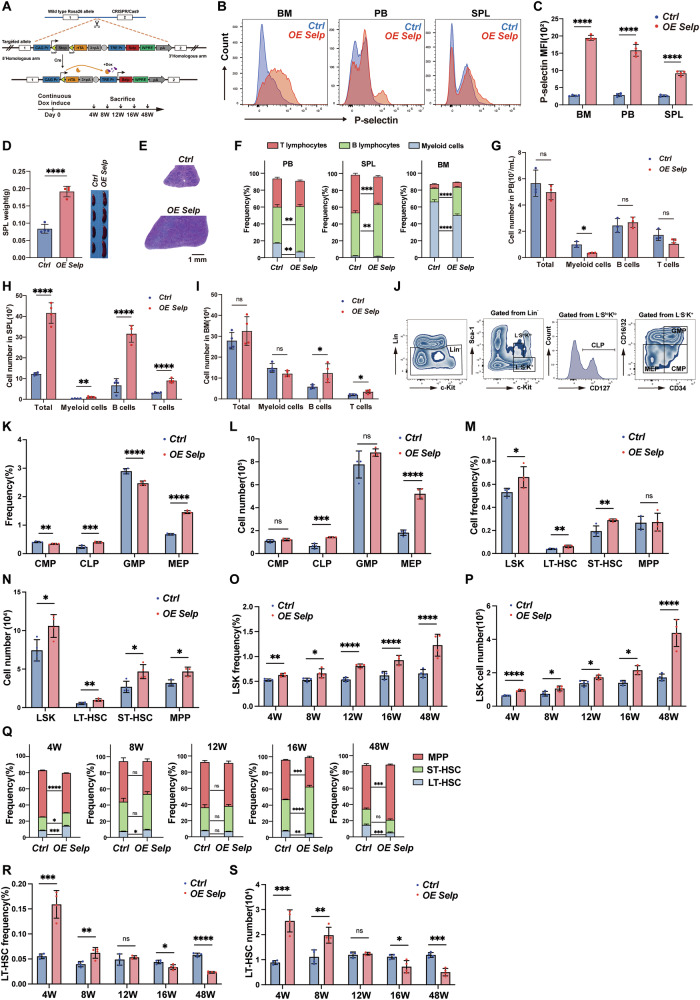
*Selp* overexpression causes a hematopoiesis disorder. **A** Schematic diagram describing the establishment of *Selp* overexpression mouse. **B** Representative flow cytometry plots showing P-selectin expression levels in BM, PB and SPL from *OE Selp* and control mice after 4 weeks Dox induction. **C** P-selectin MFI in BM, PB and SPL was then analyzed (*n* = 4 per group). **D** Gross appearances of spleen, and spleen weight. **E** Photomicrographs for H&E-stained spleen. **F** Percentage of granulocytes, B cells and T cells in PB, BM and SPL (*n* = 4 per group). **G**–**I** Number of total cells, granulocytes, B cells and T cells in PB, BM and SPL (*n* = 4 per group). **J** Gating strategy for hematopoietic progenitor cells. **K**, **L** The percentage and number of CMP, CLP, GMP and MEP in BM (*n* = 4 per group). **M**, **N** The percentage and number of LSK, LT-HSC, ST-HSC and MPP in BM (*n* = 4 per group). **O**, **P** The percentage and number of LSK after 4, 8-, 12-, 16- and 48-weeks Dox induction (*n* = 4 per group). **Q** The percentage of LT-HSC, ST-HSC and MPP after 4, 8-, 12-, 16- and 48-weeks Dox induction (*n* = 4 per group). **R**, **S** The percentage and number of LT-HSC after 4, 8-, 12-, 16- and 48-weeks Dox induction (*n* = 4 per group). Error bars denote mean ± SEM. ns, no significance, *P < 0.05, **P < 0.01, ***P < 0.001, ****P < 0.0001 (*t*-test).

To clarify the effects of high *Selp* expression on hematopoietic homeostasis, B6.*Selp*^Loxp/0^ mice crossed with B6.*Vav-Cre* mice to obtain mice with inducible *Selp* expression (B6.*Selp*^*Loxp/0*^*Vav-Cre, OE Selp*) (Fig. 3A), in which *Selp* can be specifically expressed by hematopoietic cells upon doxycycline (Dox) induction (Fig. 3B, C). Four-week Dox induction in adult *OE Selp* mice revealed systemic hematopoietic perturbations compared to control littermates. Overall, the spleen weight and volumes increased significantly in *OE Selp* mice (Fig. 3D). The histological mouse spleen sections showed no significant structural differences between *OE Selp* and control mice (Fig. 3E). Multi-compartment analysis demonstrated compartment-specific alterations. Lineage bias analysis confirmed B-lymphoid predominance across all hematopoietic compartment (Fig. 3F). Peripheral blood exhibited leukopenia with myeloid and T-cell reduction (Fig. 3G). In spleen, splenic hyperplasia coincided with pan-hematopoietic amplification, the total number of WBC increased dramatically (Fig. 3H), while bone marrow showed mild leukocyte decline with lymphoid expansion (Fig. 3I). Hematopoietic stem and progenitor profiling (Fig. 3J) uncovered fundamental shifts in differentiation trajectories. *OE Selp* mice displayed expansion of common lymphoid progenitors (CLPs) and megakaryocyte-erythroid progenitors (MEPs), contrasted by decreased common myeloid (CMP) and granulocyte-monocyte progenitors (GMP) proportions (Fig. 3K, L). For HSC, *OE Selp* mice exhibited significant increased proportions and counts in LSKs, LT-HSC, ST-HSC and MPP (Fig. 3M, N). Collectively, *Selp* overexpression enhanced hematopoiesis, drove lymphoid lineage predominance and suppressed myeloid commitment, suggesting that *Selp* may be a critical regulator of HSC fate determination.

To examine the long-term effects of *Selp* overexpression on HSC, OE *Selp* mice and control littermates were administrated with Dox in drinking water, and HSC were analyzed at 4-, 8-, 12-, 16-, and 48-week post-induction. While total HSC pools showed progressive expansion (Fig. 3O, P), primitive LT-HSC pool exhibited time-dependent depletion (Fig. 3Q–S). ST-HSC and MPP exhibited compensatory increases, indicating accelerated differentiation outputs. These effects indicated that chronic *Selp* overexpression initially drives HSC hyperproliferation, subsequently leading to LT-HSC exhaustion through overactivation.

### *Selp* overexpression impairs HSC function

**Fig. 4:**
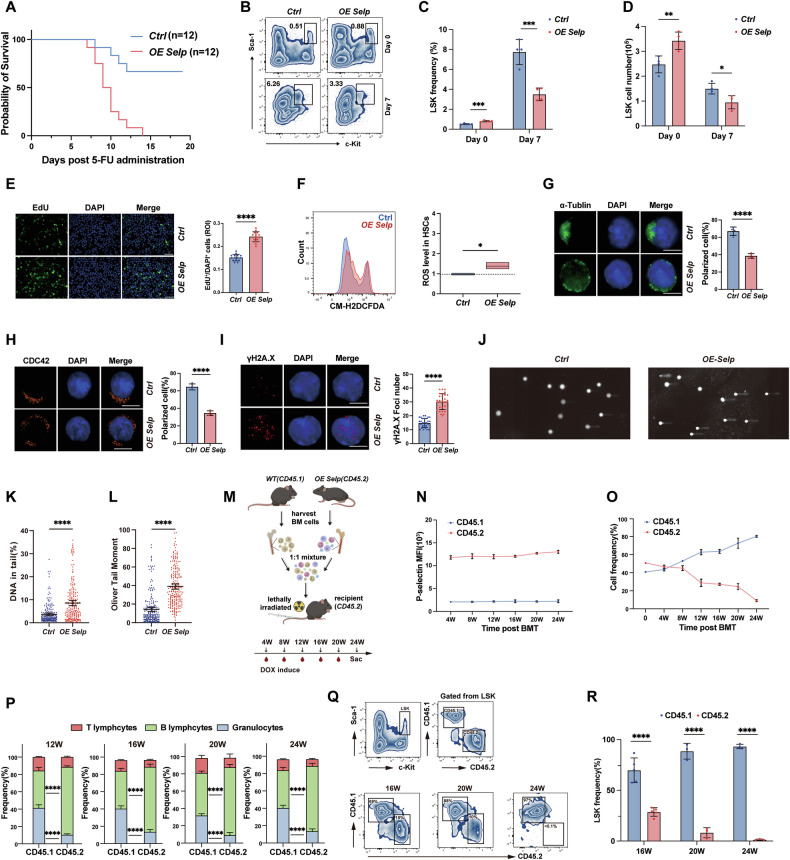
*Selp* overexpression affects the function of hematopoietic stem and progenitor cells. **A** Kaplan–Meier-style survival curves from 5-FU treated control and *OE Selp* group mice (*n* = 12 per group). **B**–**D** The percentage and number of HSC in BM from 5-FU treated control and *OE Selp* group mice at day 0 and 7 post 5-FU treatment (*n* = 4 per group). **E** Left: Representative immunofluorescence images show EdU-positive cells (green). Nuclei were counterstained with DAPI (blue). Scale bar: 50 μm. Right: The EdU incorporation rate was calculated as the ratio of EdU-positive cells to total DAPI⁺ nuclei. Each data point represents an independent region of interest (ROI). **F** Boxplots representing total ROS levels in HSC measured by CM-H2DCFDA staining. **G**, **H** Left: representative HSC isolated by FACS and stained with anti-αTubulin(green) or CDC42(orange) antibodies and DAPI (blue). Scale bar, 5 μm. Right: percentage of control and *OE Selp* HSC cells with a polar distribution of αTubulin or CDC42. Each dot represents an independent ROI. **I** Left: representative HSC isolated by FACS and stained with anti-γH2A.X antibodies (red) and DAPI (blue). Scale bar, 5 μm. Right: bar graph showing mean number of γH2A.X foci in control and *OE Selp* HSC. Each dot represents one cell. **J** Comet Assay results for HSC isolated from control and *OE Selp* mice. **K** Comet tail DNA content analysis using Comet Assay Analysis Software. Each dot represents one cell (*n* = 200 per group). **L** Olive tail moment results for control and *OE Selp* HSC. Each dot represents one cell (*n* = 200 per group). **M** Schematic diagram describing competitive repopulation assay. **N** The expression of P-selectin was examined every 4 weeks post BMT. **O** The percentage of committed cells in the PB from CD45.2 (*OE Selp*) and CD45.1 (*WT*) HSC at weeks 4-, 8-, 12-, 16-, 20- and 24-weeks post BMT. **P** Lineage differentiation analysis of peripheral blood cells from different origins in recipient mice. **Q**, **R** The percentage of replenished HSC in the BM from CD45.2 (*OE Selp*) and CD45.1 (*WT*) HSC at 16-, 20- and 24-weeks post BMT. Error bars denote mean ± SEM. *P < 0.05, **P < 0.01, ***P < 0.001, ****P < 0.0001 (*t*-test).

To determine if *Selp* overexpression activates quiescent HSC, *OE Selp* and control mice received 5-FU—a DNA synthesis inhibitor that selectively kills proliferating cells [23]. *OE Selp* mice exhibited complete mortality within 14 days versus 25% in controls (Fig. 4A). Moreover, On the day 7 post 5-FU exposure, the proportion and number of HSC were significantly lower in *OE Selp* mice (Fig. 4B–D), confirming *OE Selp* HSC were more sensitive to 5-FU-induced cytotoxicity. These results indicated that *Selp* overexpression activates quiescent HSC to an active proliferation state. enhanced HSC proliferation. To further verify whether *Selp* overexpression drives more HSC into the cell cycle, we performed an EdU incorporation assay in vivo. Remarkably, the proportion of EdU^+^ HSC in *OE Selp* mice was considerably higher in contrast to controls (Fig. 4E). Generation of reactive oxygen species (ROS) is a key event in cells following active proliferative activity [24]. Total ROS level in *OE Selp* HSC was significant increased compared to controls (Fig. 4F).

Cell polarity is an important indicator of cellular functional status and is considered a fundamental attribute of eukaryotic cells. The maintenance of cell polarity is crucial for the maintenance of HSC homeostasis. Previous studies have confirmed that tubulin and CDC42 play a significant role in cell polarity, and their polarized distributions can be used as biomarkers of cell polarity status [25]. To investigate the effect of *Selp* overexpression on cell polarity status of HSC, immunofluorescence assays for the distributions of tubulin/CDC42 were performed on *OE Selp* and control HSC. Strikingly, *OE Selp* HSC exhibited a depolarized, diffuse distribution of tubulin and CDC42, in contrast to controls, which had polarized distributions of tubulin and CDC42 (Fig. 4G, H). Genomic stability is another biomarker reflecting the functional homeostasis of HSC [26]. Furthermore, the accumulation of DNA damage is a hallmark of cellular aging [27]. To characterize the biological effects of *Selp* overexpression on genomic stability, HSPCs isolated from *OE Selp* and controls mice were examined by an γH2A.X immunofluorescence assay, which demonstrated that more γH2A.X foci occurred in *OE Selp* HSPCs (Fig. 4I), reflecting elevated DNA damage. A further comet electrophoresis assay was conducted to quantify DNA damage. As expected, *OE Selp* HSPCs exhibited severer DNA damage with more DNA fragments in the comet tail and elevated OTM compared to those from control mice (Fig. 4J–L).

To investigate whether *Selp* overexpression impairs the hematopoietic reconstitution capacity of HSC, *OE Selp* (CD45.2) and *WT* (B6.SJLPtprcaPepcb/BoyJ, CD45.1) BM cells were mixed equally and transplanted into lethally irradiated CD45.2 mice (Fig. 4M). The expression of P-selectin (Fig. 4N) and donor-derived cells (Fig. 4O) in PB were examined at 4-, 8-, 12-, 16-, 20- and 24-weeks post BMT. Overall, BM cells from *OE Selp* mice had a weaker capacity for hematopoietic repopulation (Fig. 4O). Peripheral lineage analysis revealed skewed differentiation in *OE Selp* versus control, with increased B lymphocytes and decreased granulocytes (Fig. 4P). HSC were analyzed at 16-, 20-, and 24-weeks post BMT, and *OE Selp* donor-derived HSC exhibited progressive depletion in recipient bone marrow, plummeting from 15% at week 16 to less than 0.1% by week 24 (Fig. 4Q, R). Taken together, these findings demonstrated that *Selp* overexpression not only impairs HSC long-term multilineage reconstitution but also disrupts lineage commitment, skewing differentiation toward B-lymphocytes at the expense of myeloid potential.

### High expression of *Selp* causes transcriptional changes in HSC

**Fig. 5:**
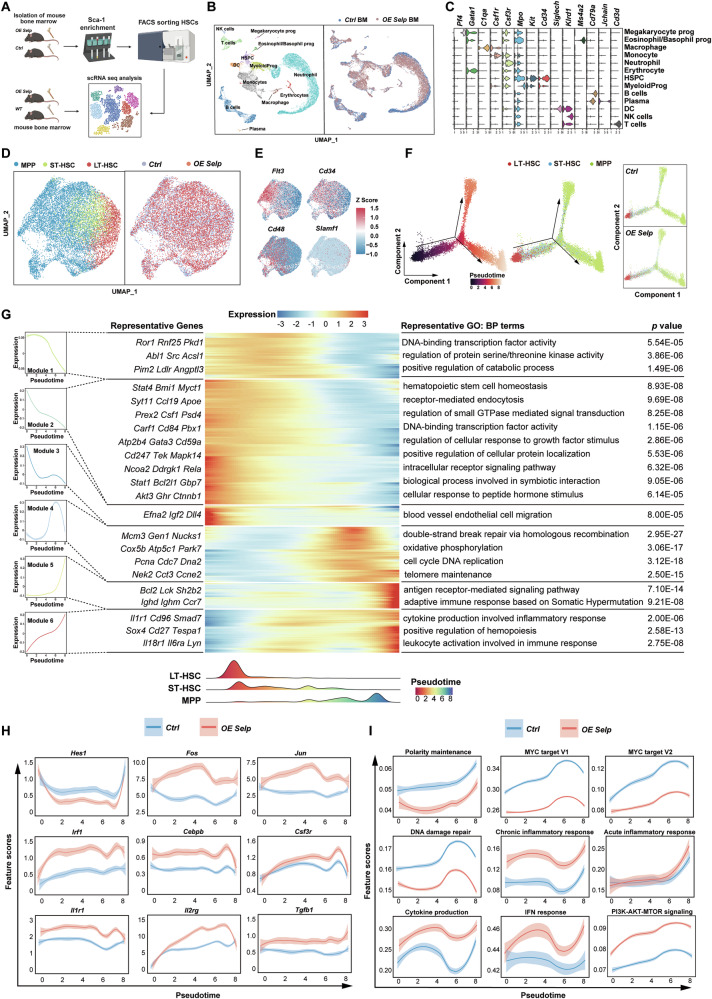
Analysis of transcriptional changes in *Selp* overexpression HSC. **A** Schematic overview of scRNA analysis. **B** Left: Cell clusters in BM cells were visualized using UMAP. Colors indicate cell types. Each dot represents one cell. Right: Cell distribution for control and *OE Selp* group. **C** Violin plots showing the expression of specific marker genes in each cell cluster. **D** Left: Cell clusters in isolated HSCs were visualized using UMAP. Colors indicate cell types. Each dot represents one cell. Right: Cell distribution for control and *OE Selp* group. **E** UMAP showing the expression of specific marker genes in each cell cluster. **F** Pseudotime-ordered analysis of HSC from the control and *OE Selp* samples, and 2D graph of the pseudotime-ordered HSC from control and *OE Selp* samples. **G** Heat map showing dynamic changes in gene expression along the pseudotime (cataloged hierarchically into six gene modules). Adjusted *p* value < 0.05 was considered statistically significant for Gene Ontology (GO) enrichment analysis. **H** Two-dimensional plots showing the dynamic expression of significantly enhanced (*Fos, Jun, Irf1, Cebpb, Csf3r, Il1r1, Il2rg, Tgfb1*) and reduced (*Hes1*) genes in *OE Selp* compared with control along the pseudotime. **I** Two-dimensional plots showing the dynamic expression of scores for abnormality of polarity maintenance, DNA damage repair, inflammatory response, cytokine production, IFNγ response, PI3K-AKT-MTOR signaling and MYC target along with the pseudotime in control and *OE Selp* groups. The values of the y axis are the calculated GSVA scores. Pathways are selected from the GSEA enrichment results in *OE Selp*. (NES > 1, NOM *p* val < 0.05, and FDR *q* val < 0.25).

To investigate the hematopoietic transcriptional landscape and determine the alterations in *OE Selp* HSPCs, single-cell RNA-seq of whole BM cells and isolated HSC from *OE Selp* mice and control mice was performed with the 10X Chromium platform (Fig. 5A). Following the implementation of quality control, a total of 38941 (*OE Selp* BM, *n* = 11449; control BM, *n* = 8674; *OE* HSC, *n* = 9092; control HSC, *n* = 9726) single-cell profiles were included in the downstream analyses. Dimensional reduction using uniform manifold approximation and projection (UMAP) demonstrated the effective integration of the datasets from different samples. For BM cells, the cell clusters were manually annotated into 13 distinct cell types (Fig. 5B) with unique gene expression patterns (Fig. 5C). These populations included HSPCs, megakaryocyte progenitors, eosinophil/basophil progenitors, myeloid progenitors, B lymphocytes, T lymphocytes, dendritic cells (DC), natural killer cells (NK), neutrophils, macrophages, monocytes, plasma cells and erythrocytes. For isolated HSPCs, the cell clusters were manually annotated into three distinct cell types (Fig. 5D) with specific‌ gene expression patterns (Fig. 5E). These populations included LT-HSC, ST-HSC, MPP. The cell cluster results demonstrated no evident disparities in cell subpopulations within either BM samples or isolated HSPCs samples between the *OE Selp* and control groups (Fig. 5B, D). In contrast to the stepwise progression observed in other cell types, HSC undergo a continuous differentiation process. To further elucidate the progression of this differentiation process, we performed pseudotime analysis on HSC scRNA sequencing data. The cells were ordered using Monocle to model the potential trajectory of HSC transitioning from LT-HSC, ST-HSC to MPP. The left side of the trajectory was identified as LT-HSC, and the right furcation of the trajectory was identified as MPP. It was observed that *Selp* overexpression appeared to promote the rapid differentiation of LT-HSC into ST-HSC and MPP rather than maintaining the self-renewing stem cell pool (Fig. 5F).

Six different gene expression modules were identified on the basis of transcriptional changes associated with transitional states (Fig. 5G). An evaluation was conducted in which the single-gene transcriptional changes of *OE Selp* HSC were compared with those of control HSC along the pseudotime (Fig. 5H). During the course of pseudotime, the observations revealed a decrease in the expression levels of *Hes1* and *Myc*, which participated in preventing stem cells from prematurely differentiation, thereby maintaining their stemness. Furthermore, multiple genes involved in inflammatory responses—including *Fos, Jun, Csf3r, Cebpb, Il1ra, and Il2rg*—exhibited significant upregulation. Moreover, gene set variation analysis (GSVA) scores of pathways were calculated on the scale of pseudotime. The results demonstrated that *OE Selp* HSC exhibited elevated scores for DNA damage repair and inflammatory response, while the scores for polarity maintenance and *Myc*-targeted pathways were notably reduced (Fig. 5I). Together, we concluded that *Selp* overexpression tends to promote the rapid differentiation of LT-HSC into ST-HSC and MPP. Moreover, the inflammatory response and transcriptional states differed considerably between *OE Selp* and control HSC, suggesting that inflammatory-induced activation of HSC should be considered for understanding the effects of *Selp* on HSC.

### *Selp* affects HSC homeostasis through inflammatory response receptors

**Fig. 6:**
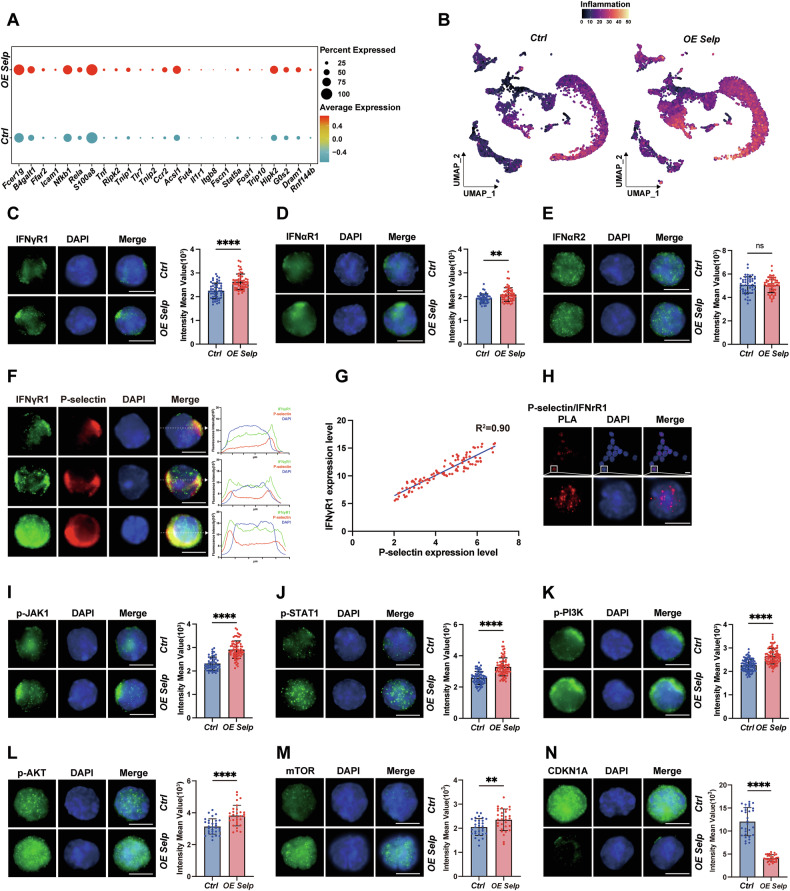
*Selp* affects HSC homeostasis through inflammatory response receptors. **A** Gene set used for calculating inflammation response pathway signature score. **B** UMAP visualization showing the differences in the inflammation response pathway signature score among BM cells of control and *OE Selp* group. **C**–**E** Left: representative HSC isolated by FACS and stained with anti-IFNγR1 (green), IFNαR1 (green) or IFNαR2 (green) antibodies and DAPI (blue). Scale bar, 5 μm. Right: Quantification of IFNγR1, IFNαR1 or IFNαR2 in the control group and *OE Selp* HSC. Each dot represents one cell (*n* = 30–50 per group). **F** Representative distribution of P-selectin (red) and IFNγR1 (green) in HSC with different P-selectin expression levels. Nuclei are stained with DAPI (blue). Scale bar, 5 μm. Relative fluorescence intensity plot obtained by collecting pixel intensity through the section of the relative cell are also shown. **G** Correlation analysis of P-selectin and IFNγR1 expression level in HSC. **H** Proximity ligation assay was used to detect protein-protein interactions in fixed cells. After incubated with P-selectin and IFNγR1, cells were then incubated with the PLA probes, ligation and amplification were performed according to the manufacturer’s instructions. **I**–**N** Left: representative HSC isolated by FACS and stained with anti-p-JAK1 (green), p-STAT1 (green), p-PI3K (green), p-AKT(green), mTOR (green) or CDKN1A (green) antibodies and DAPI (blue). Scale bar, 5 μm. Right: Quantification of p-JAK1, p-STAT1, p-PI3K, p-AKT, mTOR or CDKN1A in the control and *OE Selp* HSC. Each dot represents one cell (*n* = 30–50 per group). Error bars denote mean ± SEM. ns no significance, **P < 0.01, ****P < 0.0001 (*t*-test).

As the GSVA scores of pathways on the scale of pseudotime suggested that genes involved in “IFN response” were enriched and increased in *OE Selp* HSC (Fig. 5K). These results indicated the presence of a synergistic positive feedback regulatory mechanism between inflammation and *Selp*. Specifically, inflammation induces an increase in *Selp* expression, and conversely, high *Selp* expression itself also leads to an inflammatory response in HSPCs. Further, the inflammatory response in BM cells was assessed with distinct gene expression patterns (Fig. 6A). An elevated inflammatory response was observed in the BM cells of the *OE Selp* group in comparison with the control group (Fig. 6B).

In the subsequent research, a range of IFN-related receptors were analyzed. The results demonstrated that the IFNγR1 was significantly increased (Fig. 6C). While IFNαR1 showed a less but significant elevation (Fig. 6D), IFNαR2 expression remained unchanged (Fig. 6E). To ascertain whether there was a correlation between the co-localization of IFNγR1 and P-selectin, an immunofluorescence assay was performed in *OE Selp* HSC. It was determined that IFNγR1 and P-selectin displayed a polarized co-localized distribution on the HSC surface (Fig. 6F). Furthermore, an analysis of HSC exhibiting varying P-selectin expression levels confirmed a concomitant linear relationship between IFNγR1 and P-selectin levels within the HSC (Fig. 6G). Proximity ligation assays (PLA) were subsequently performed in *OE Selp* HSC to assess the correlation between observed colocalizations and close molecular interactions. Specific PLA signals indicating direct proximity between P-selectin and IFNγR1 were detected (Fig. 6H). Comprehensive analysis of IFNγR1 downstream signaling cascades [28–32] demonstrated significantly elevated phosphorylation levels of JAK1, STAT1, PI3K, and AKT in *OE Selp* HSC versus controls (Fig. 6I–L). This hyperactivation was further evidenced by increased mTOR protein abundance (Fig. 6M) and reduced p21 expression (Fig. 6N), aligning with enhanced proliferative signaling and attenuated cell-cycle arrest. Critically, mTOR activation drives dormant HSC into rapid cycling but ultimately impairs hematopoiesis and self-renewal—a process mediated by elevated mitochondrial metabolism and ROS production. Increased ROS levels (Fig. 4F) trigger DNA damage accumulation (Fig. 4J), while compromised DNA repair capacity (Fig. 5I) further exacerbates functional decline in HSC. In order to biologically validate the effects of IFNγ signaling pathways on HSC proliferation, 5-FU-treated BM cells were harvested and cultured with INF-γ. The results demonstrated that INF-γ could significantly promote HSC proliferation with simultaneously upregulated P-selectin expression level (Supplementary Fig. 2). These results provide a comprehensive explanation for the phenomena observed in *OE Selp* mice. Specifically, elevated expression levels of *Selp* lead to the activation of INF-γ responses, resulting in a greater number of HSC undergoing proliferation and differentiation. With such chronic stimuli, the function of HSC was eventually impaired.

### High expression of *Selp* diminishes the pathogenic capacity of leukemia stem cells

**Fig. 7:**
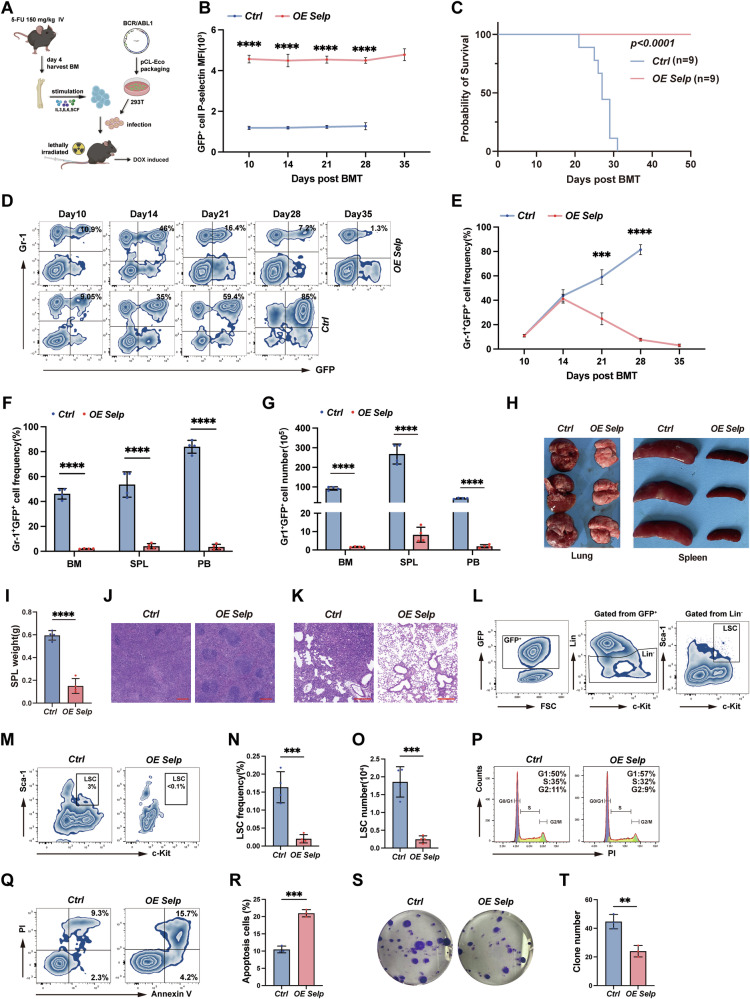
*Selp* overexpression inhibits the progression of *BCR-ABL1* induced CML. **A** Schematic diagram describing *BCR-ABL1* induced CML mouse model. **B** P-selectin MFI in PB GFP^+^ cells from control and *OE Selp* group recipients. **C** Kaplan–Meier-style survival curves for control and *OE Selp* group recipients. **D**, **E** GFP^+^Gr-1^+^cells percentages in PB for all recipients induced (day 10, 14, 21, 28 and 35 post BMT). **F**, **G** Percentage and number of GFP^+^Gr-1^+^ cells in BM, spleen and PB. **H** Gross appearances of lung and spleen from each group. **I** Spleen weight of each group recipients (*n* = 4 per group). **J**, **K** H&E staining of spleen and lung sections from recipients receiving *BCR-ABL1* transduced control or *OE Selp* BM. **L** Gating strategy of LSC. Percentage (**M**, **N**) and number (**O**) of leukemia stem cells (LSC, GFP^+^Lin^-^Sca-1^+^c-Kit^+^) in BM from recipients receiving *BCR-ABL1* transduced control or OE Selp BM (*n* = 4 per group). **P** The normal LSK frequence in GFP^-^ population (*n* = 4 per group). **Q** The cell cycle analysis for isolated LSC from each group. **R**, **S** LSC apoptosis within BM of recipient mice. **T**, **U** Colony formation assay. Sorted *BCR-ABL1*-expressing LSCs from recipient mice BM were seeded into 6 well plates containing 2 mL M3234 medium, the total number of colonies were counted after 7 days, representative data displayed. Error bars denote mean ± SEM. *P < 0.05, **P < 0.01, ***P < 0.001, ****P < 0.0001 (*t*-test).

In consideration of the aforementioned results, sustained *Selp* overexpression drives HSC functional exhaustion through chronic inflammatory stimuli. To determine whether elevated *Selp* expression impairs the leukemogenic potential of leukemia stem cells (LSC), we utilized a *BCR-ABL1*-transduced chronic myeloid leukemia (CML) mouse model. BM cells from 5-FU-treated *OE Selp* or control mice were harvested and stimulated with cytokines and infected with retrovirus to ectopically express *BCR-ABL1* oncogenes in HSC. Subsequently, those cells were transplanted intravenously into lethally irradiated recipient mice (Fig. 7A). Detection of P-selectin expression levels on GFP⁺ cells in the PB of recipient mice confirmed that sustained Dox treatment specifically induced high P-selectin expression on donor-derived GFP⁺ blood cells in the *OE Selp* group (Fig. 7B). Survival analysis revealed that all control mice succumbed to aggressive CML progression within 31 days post-transplantation, whereas 100% of *OE Selp* recipients survived throughout the 50-day observation period (Fig. 7C). Peripheral tumor burden monitoring showed no significant difference in the proportion of GFP⁺Gr-1⁺ cells between *OE Selp* and control groups within the first 14 days. However, beyond day 14, the tumor burden in the *OE Selp* group exhibited sustained reduction, while controls showed progressive escalation (Fig. 7D, E). This demonstrates that *Selp* overexpression does not prevent disease initiation but significantly impedes its progression.

To evaluate systemic leukemia burden, four recipients from each group at day 21 post-BMT were sacrificed and assessed tumor dissemination across major hematopoietic organs. Consistently, *OE Selp* recipients exhibited significantly reduced leukemia burden in BM, SPL and PB, with diminished proportions and absolute counts of tumor cells (Fig. 7F, G). Concurrently, control spleens exhibited diffuse enlargement, and lungs showed dense hemorrhagic foci. In contrast, *OE Selp* spleens were only mildly enlarged with no apparent pulmonary hemorrhage (Fig. 7H, I). Furthermore, there was less infiltration of leukemia cells into the lungs and spleens (Fig. 7J, K). Leukemia stem cell (LSC) analysis revealed that *OE Selp* recipients had significantly decreased proportions (Figure L-N) and absolute numbers (Fig. 7O) of LSC versus controls. Analysis of normal LSK frequence in GFP^-^ population showed slight decrease in *OE Selp* group (Fig. 7P), which suggested that LSC were more sensitive to *Selp* overexpression induced depletion. We further analyzed the cell cycle of LSC and found that overexpression of *Selp* resulted in a G0/G1 phase accumulation and a concomitant reduction in S + G2/M phase cells (Fig. 7Q). The LSC viability analysis demonstrated significantly enhanced apoptosis in *OE Selp* LSC compared to controls (Fig. 7R, S). An in vitro colony forming assay showed that *OE Selp* LSC generated significantly fewer colonies than control LSC (Fig. 7T, U). Collectively, these results indicated that high *Selp* expression impaired the self-renewal capability of LSC.

## Discussion

P-selectin, a transmembrane glycoprotein belonging to the selectin family of cell adhesion molecules, is canonically expressed on activated endothelial cells and platelets to mediate leukocyte rolling during inflammatory responses [15]. Previous studies have demonstrated that P-selectin plays a critical role in immune cell adhesion [33]. Furthermore, E-selectin, a member of selectin family, has been reported to regulate HSC dormancy and self-renewal. HSC quiescence was enhanced, and self-renewal potential was increased in E-selectin knockout (*Sele*^*−/−*^) mice [34]. However, the role of P-selectin in homogenesis and HSC remains to be elucidated. Our findings established P-selectin as a central mediator of HSC dysfunction during aging and inflammation. Through integrated single-cell transcriptomics and functional validation, we identified *Selp* as a consistently upregulated gene in aged long-term HSC (LT-HSC) across multiple independent datasets. This transcriptional elevation correlates with a broader inflammatory signature characterized by hyperactivation of TNF-α, IFN-γ, and IL-2/STAT5 signaling and suppression of DNA repair mechanisms. Besides, P-selectin expression was dynamically amplified under diverse inflammatory stressors (sepsis, pneumonia, asthma) in HSC (Fig. 2L, R, X). In transgenic mouse models, *Selp* overexpression results in HSC in homeostasis disruption. Phenotypically, *Selp* overexpression led to a continuous increase in HSC percentage and absolute numbers within the mouse bone marrow (Fig. 3M–P), while the LT-HSC pool gradually depleted over time with prolonged overexpression (Fig. 3Q–S). Further analysis confirmed that HSC quiescence was disrupted in *Selp* overexpression mice, evidenced by hypersensitivity to 5-FU-induced cytotoxicity (Fig. 4A–D) and increased EdU incorporation (Fig. 4E). This is accompanied by elevated ROS production (Fig. 4F), compromised polarity marked by diffuse tubulin/CDC42 distribution (Fig. 4G,H), increased DNA damage accumulation (Fig. 4I–L). *Selp* overexpression impaired long-term HSC capacity of self-renewal and blood-lineage replenishment under a competitive repopulation condition.

scRNA analysis showed elevated inflammatory responses in both whole BM (Fig. 6A, B) and isolated HSC samples (Fig. 5I). Pseudotemporal pathway activity analysis confirmed that *Selp* overexpression led to downregulation of genes associated with polarity maintenance, MYC target pathways and DNA damage repair, and upregulation of pathways related to chronic inflammatory responses, cytokine production, IFN responses and PI3K-AKT-MTOR signaling (Fig. 5I). These results suggest the mechanistic cascade linking *Selp* to HSC exhaustion involves in activation of inflammatory responses. Further immunofluorescence analysis demonstrated that P-selectin co-localizes with IFNγR1 on the HSC surface (Fig. 6F), and proximity ligation assay confirmed direct interaction of P-selectin and IFNγR1 (Fig. 6H), which driving activation of JAK1-STAT1 and PI3K-AKT-mTOR signaling axes (Fig. 6I–M). Aberrant PI3K-AKT-MTOR drives many pathways to increase ROS levels through directly modulating mitochondrial bioenergetics and activating NADPH oxidases (NOXs) or indirectly producing ROS as a metabolic by-product. ROS exposure can cause oxidative damage to DNA, leading to the formation of single- or double-strand breaks (DSB) in severe circumstances [35]. Additionally, in *OE Selp* HSC, impaired DNA damage repair capacity (Fig. 5I) results in greater accumulation of DNA damage (Fig. 4I–L). Eventually, the chronic activation of JAK1-STAT1-PI3K-AKT-mTOR signaling axes result in the LT-HSC exhaustion.

Sustained P-selectin overexpression significantly impaired the leukemogenic potential of LSCs in a *BCR-ABL1* CML mouse model. While *Selp* overexpression did not prevent disease initiation, it potently blocked progression. All control mice succumbed within 31 days, whereas 100% of *OE Selp* recipients survived the 50-day observation (Fig. 7C). Tumor burden was initially comparable, but diverged markedly after day 14, showing sustained reduction only in the *OE Selp* group (Fig. 7D, E). Systemic evaluation revealed significantly reduced leukemia burden in bone marrow, spleen, and blood of *OE Selp* mice, along with mitigated organ pathology (splenomegaly, hemorrhage) and reduced tumor infiltration (Fig. 7F–K). Crucially, *OE Selp* LSCs exhibited decreased numbers (Fig. 7M–O), G0/G1 cell cycle arrest (Fig. 7P), increased apoptosis (Fig. 7Q, R), and severely impaired colony-forming capacity (Fig. 7S, T). These results demonstrate that *Selp* overexpression compromises LSC function by inducing cell cycle arrest, promoting cell death, and abrogating self-renewal. Moreover, LSC were more sensitive to *Selp* overexpression induced depletion compared to normal HSC, precise regulation of Selp expression level could therefore selectively eliminate LSCs while minimizing detrimental effects on normal HSC. Our data demonstrate that sustained *Selp* overexpression drives HSC exhaustion and impairs LSC function through cell cycle arrest, apoptosis, and loss of self-renewal. This phenotype may exhibit contextual sensitivity, particularly across inflammatory conditions such as acute tissue damage versus chronic microenvironmental stress where dynamic selectin ligand interactions could modulate outcomes. While E-selectin is constitutively expressed in bone marrow niches and L-selectin mediates leukocyte rolling, potential compensation or synergy between these adhesion receptors remains uncharacterized in our model. This study focused primarily on the direct impact of sustained *Selp* overexpression on LSC and HSC function, rather than its potential effects on the bone marrow niche. The consequences of *Selp* dysregulation within the niche microenvironment and its contribution to the observed LSC depletion and HSC exhaustion remain unexplored and represent an important avenue for future investigation into the broader mechanisms of selectin-mediated stem cell regulation.

## Material and method

### Ethics approval and consent to participate

All animal experiments were performed in accordance with guidelines approved by the Institutional Animal Care and Use Committees of Sichuan University (Approval ID: 20250227043), with protocols approved by the Animal Care and Use Committee of the State Key Laboratory of Biotherapy, Sichuan University. This study does not contain any human sample.

### Mice

Recipient C57BL/6 (CD45.2) mice purchased from Charles River (Sichuan, CN) and maintained at the State Key laboratory of Biotherapy Animal Center. B6.Vav-Cre mice were gifted by Cyagen (Jiangsu, China). B6.SJL (CD45.1) mice (Cat. NO. NM-KI-210226) were purchased from Shanghai Model Organisms Center, Inc. To generate mice with tissue-specific inducible Selp expression (B6.Selp^mut/+^), a regulatory cassette carried a loxp-flanked “stop” sequence, a reverse tetracycline transactivator (rtTA) and a tetracycline response element (Tre)-regulated promoter was inserted between the CAG promoter and the first exon of the *Selp* gene, and a Rosa26 allele element was knocked in(Figure). Then, B6.*Selp*^mut/+^ mice were crossed with B6.Vav-Cre mice to obtain B6.*Selp*^mut/+^Vav-Cre mice. The Cre recombinase removed the stop sequence resulting in rtTA expression. With continuous administration of Doxycycline (DOX) in drinking water at 250 μg/mL, Tre-regulated promoter initiated Selp expression. Age and gender-matched littermates with a *Selp*^mut/+^ genotype were used as controls and treated with the same conditions as B6.*Selp*^mut/+^ Vav-Cre mice.

### Flow cytometry and cell sorting

White blood cell form donor mice peripheral blood (PB), spleen (SPL) and bone marrow (BM) were incubated in red blood lysis buffer to lyse erythrocytes. Cell immunostaining was performed according to standard procedures. The cellular composition in these organs was determined by flow cytometry and analyzed via FlowJo software (V10.10). HSC and progenitors were categorized using the following markers: LSKs: Lin^-^Sca-1^+^c-Kit^+^; LT-HSC: Lin^-^Sca-1^+^c-Kit^+^CD48^-^CD150^+^; ST-HSC: Lin^-^Sca-1^+^c-Kit^+^CD48^-^CD150^-^; MPP: Lin^-^Sca-1^+^c-Kit^+^CD48^+^CD150^-^; CMPs: Lin^-^Sca-1^-^c-Kit^+^CD16/32^-^CD34^+^; GMPs: Lin^-^Sca-1^-^c-Kit^+^CD16/32^+^CD34^+^; MEPs: Lin^-^ Sca-1^-^c-Kit^+^CD16/32^-^CD34^-^; CLPs: Lin^-^Sca-1^low^c-Kit^low^CD127^+^; B cells: B220^+^; T cells: CD3^+^; myeloid cells: Gr-1^+^. To isolate HSC, Sca-1^+^ cell enrichment was performed to enrich for Sca-1 positive cells. Lineage, c-Kit dual-positive cells were then stained as aforementioned and sorted using a BD FACS Aria II or a BD FACS Aria III.

### Sepsis mouse model

8-week-old adult male C57BL/6 mice were used in this study. In the sepsis group, mice were anesthetized with isoflurane. After disinfecting the abdominal skin, a small incision was made in the lower abdomen. Cecal perforation was performed, followed by suturing of the wound. Postoperatively, the mice were placed on a heating pad for recovery. Sham surgery was performed on the control group mice. 24 h post-surgery, mice from both groups were sacrificed for HSC analysis.

### Pneumonia mouse model

8-week-old adult male C57BL/6 mice were used in this study. In the sepsis group, mice were anesthetized with pentobarbital intraperitoneally and placed in a supine position when they reached a stage of deep, rapid breathing. During inhalation, a 10 μl pipette tip was used to administer the drug solution in small, multiple doses via nasal instillation, allowing the liquid to enter the lungs through the airways and directly stimulate the bronchi and lung tissue. The control group received saline solution via the same procedure. 48 h post-surgery, mice from both groups were sacrificed for HSC analysis.

### Asthma mouse model

Ovalbumin (Sigma, St. Louis, MO) was dissolved in PBS at a concentration of 100 µg/100 µl, and an equal volume of Imject™ Alum (Thermos Scientific) was then added. The sensitization injection should be configured in a 1:1 ratio. It is essential to vortex the Imject™ alum with the ovalbumin solution when dripping, and to maintain this mixing process for a period of 30 min following the addition. Ovalbumin was dissolved in physiological saline to form a nebulization-inducing solution with a volume fraction of five percent.

On days 0, 7, and 14, mice in all the model groups were administered an ovalbumin sensitizing solution intraperitoneally at a dose of 3 mg/kg body weight. Mice in all the control groups were injected with the same volume of PBS solution. From day 21 to day 25, mice in all the model groups were continuously nebulized with the induction solution for a period of 30 minutes each day, while mice in all the control groups were nebulized with normal saline for the same duration. The Montelukast-treated mice must be administered a Montelukast solution (50 mg/kg) via intragastric administration 30 minutes prior to nebulization. Twenty-four hours after the final nebulization, the mice were sacrificed for analysis.

### Immunofluorescence staining

Freshly sorted HSC were fixed with 4% PFA (Biosharp) at room temperature for 1 h. After fixation cells were seeded onto fibronectin-coated glass slides and gently washed with PBS, then permeabilized with 0.2% Triton X-100 (Sigma) in PBS for 20 min and blocked with 5% BSA (Sigma) for 30 min. Primary antibodies incubations were performed over night at 4 °C. Secondary antibodies incubations were performed for 1 h at room temperature. Samples were imaged with an AxioImager 2 microscope(Zeiss) equipped with a 63× PH objective. Images were analyzed with Zen software. The primary antibodies included: α-Tubulin (CST, #2144), CDC42(Santa, sc-8401), γH2A.X(CST, #2577), IFNγR1 (Huabio, ER63732), IFNαR1 (Huabio, EM1701-46) or IFNαR2 (Huabio, HA500284), P-Selectin (Santa, sc-8419), IL6Rα (Santa, sc-374259), p-PI3K (Huabio, HA721672), p-JAK1 (Huabio, ET1607-34), p-STAT1 (CST, #9167), p-AKT (CST, #4060).

### Comet assay

Freshly sorted HSC were centrifuged and resuspend to 1 × 10^5^/mL. Then, 5 μl HSC were combined with 50 μl previously melted LMAgarose(cooled in 37 °C water bath) and immediately pipetted onto CometSlide^TM^. Afterward, the slides were placed at 4 °C for 10 min. The slides were immersed in Lysis Solution for 30 min (or overnight). Then, the slides were immersed in 50 mL freshly prepared Alkaline Unwinding Solution(NaOH 0.4 g, 200 mM EDTA 250 μl, ddH_2_O 49.75 mL) for 20 min at room temperature. Alkaline electrophoresis(AES: NaOH 8 g, 500 mM EDTA, 2 mL; ddH_2_O up to 1 L) were performed at 21 V for 30 min. The slides were washed twice in ddH_2_O for 5 minutes each, then in 70% ethanol for 5 min. DNA staining was performed with diluted SYBR Gold for 30 min. Slides were imaged with an AxioImager 2 microscope(Zeiss) equipped with a 20× objective. Images were analyzed with Comet Analysis Software.

### Competitive repopulation assay

BM cells from B6.Selp^mut/+^ Vav-Cre(CD45.2) mice and B6.SJL(CD45.1) mice were harvested and mixed at a 1:1 ratio. The mixed BM cells were subsequently transplanted into C57BL/6J(CD45.2) recipient (1 × 10^6^ cells per mouse), which received two doses of 5 Gy of X-ray irradiation separated by 3 h. Doxycycline(DOX) was administrated in drinking water at 250 μg/mL. The repopulating capacity in peripheral blood(PB) and the percentages of donor derived HSC in the recipient BM were analyzed at multiple time points post bone marrow transplantation.

### Single-cell RNA sequencing data alignment and quality control

Raw data were assembled using Illumina’s fastq converter. After initial quality control, adaptor sequences, sequences with over three uncertain nucleotides (designated as N) and low-quality reads were removed. Only clean data for high-quality reads were aligned to the human reference genome with the 10x Genomics Cell Ranger pipeline using default parameters. Unique molecule identifiers (UMIs) for each gene and each cell barcode were counted and added to gene expression matrices. Doublets detected by Scrublet. Doublet clusters expressing markers of more than two major cell lineages, and cells meeting the following criteria were removed: (1) number of genes detected per cell <200 or >5000; (2) UMIs detected per cell over 200; (3) counts of mitochondrial genes constituting over 12% of all gene counts; and (4) counts of red blood cell genes constituting over 0.3% of all gene counts.

### Dimensionality reduction and annotation of major cell clusters

The gene expression matrices of each sample were analyzed and initially clustered for major cell subsets. Briefly, normalization and dimensionality reduction were performed on integrated matrices of isolated HSC and BM cells using the Seurat package. The batch effects in the study samples were removed by the RunHarmony function in the Harmony package. The uniform manifold approximation and projection (UMAP) method was applied to visualize a 2D projection of cell clusters from an SNN graph. Cells were clustered with highly variable genes at 0.7 resolution. The top 30 differentially expressed genes (DEGs) of each cluster were identified and carefully examined for well recognized marker genes.

### Differential expression analysis and enrichment analysis

The FindAllMarkers function implemented in Seurat was used to identify DEGs in each cluster. GO term enrichment and KEGG pathway analysis were performed by ClusterProfiler R package (version 4.0.5).59 GSVA scores were calculated by GSVA R package (version 1.34.0).60 GSEA software (version 4.1.0) 61 was used to calculate the distribution of gene sets in lists of genes ordered by population expression differences. Normalized enrichment score (NES), nominal *P* value, and false discovery rate (FDR) *q* value were used for the statistical analysis. The pathways with |NES|>1, NOM *p* value < 0.05, and FDR *q* value < 0.25 were considered to be meaningful.

### BCR-ABL1 induced CML mouse model

Donor mice were pretreated with 150 mg/kg 5-fluorouracil(5-FU) intravenously. Four days later, bone marrow cells were harvested from femurs, tibias and hipbone, and cultured for 24 h with stimulating medium containing IL-3 (10 μg/mL), IL-6 (10 μg/mL) and SCF (50 μg/mL). The BM cells were subjected to two rounds of co-sedimentation with MSCV-BCR-ABL1-IRES-eGFP retroviral stock at 1000 × *g* for 90 min at 37 °C. The transduced BM cells were subsequently transplanted into C57BL/6J recipient (5 × 10^5^ cells per mouse), which received two doses of 5 Gy of X-ray irradiation separated by 3 h. Leukemia cells (GFP^+^Gr-1^+^) in peripheral blood (PB) and leukemia stem cells(LSC, GFP^+^Lin^-^Sca-1^+^c-Kit^+^) in bone marrow(BM) were analyzed using FACS in the recipients at multiple time points post bone marrow transplantation.

### H&E staining of histology

The spleen and lung were fixed in 4% paraformaldehyde for 24 h at room temperature, followed by an overnight rinse in water. Sections were stained with H&E using standard methods. All images were obtained using a digital pathology scanner (3D HISTECH) and imported into Case Viewer software as a series of tagged image files. The representative pictures were taken at a 20× or 50× magnification.

### Statistical analysis

The results from the experiments described herein were presented using the mean plus/minus the standard error of the mean (±SEM). The statistical significance was determined using a one-way analysis of variance (ANOVA) or Student’s *t* test using Prism10. A *P* value equal to or less than 0.05 was used as the threshold when considering the statistical significance of differences.

## Supplementary information

Supplementary

## Data Availability

The raw sequence data reported in this paper have been deposited in the Genome Sequence Archive (Genomics, Proteomics & Bioinformatics 2021) in National Genomics Data Center (Nucleic Acids Res 2022), China National Center for Bioinformation / Beijing Institute of Genomics, Chinese Academy of Sciences (GSA: CRA022361) that are publicly accessible at https://ngdc.cncb.ac.cn/gsa.
