# Supplementary material for: P-selectin overexpression impairs hematopoietic stem cell homeostasis via inflammatory receptor-mediated proliferation and differentiation
Source: Cell Death Dis. 2025 Oct 21;16(1):745. doi: 10.1038/s41419-025-08050-9 (PMC12540768; doi:10.1038/s41419-025-08050-9)
Supplement: Supplementary file 1 — Supplementary [file 41419_2025_8050_MOESM1_ESM.docx]

# Supplementary

**
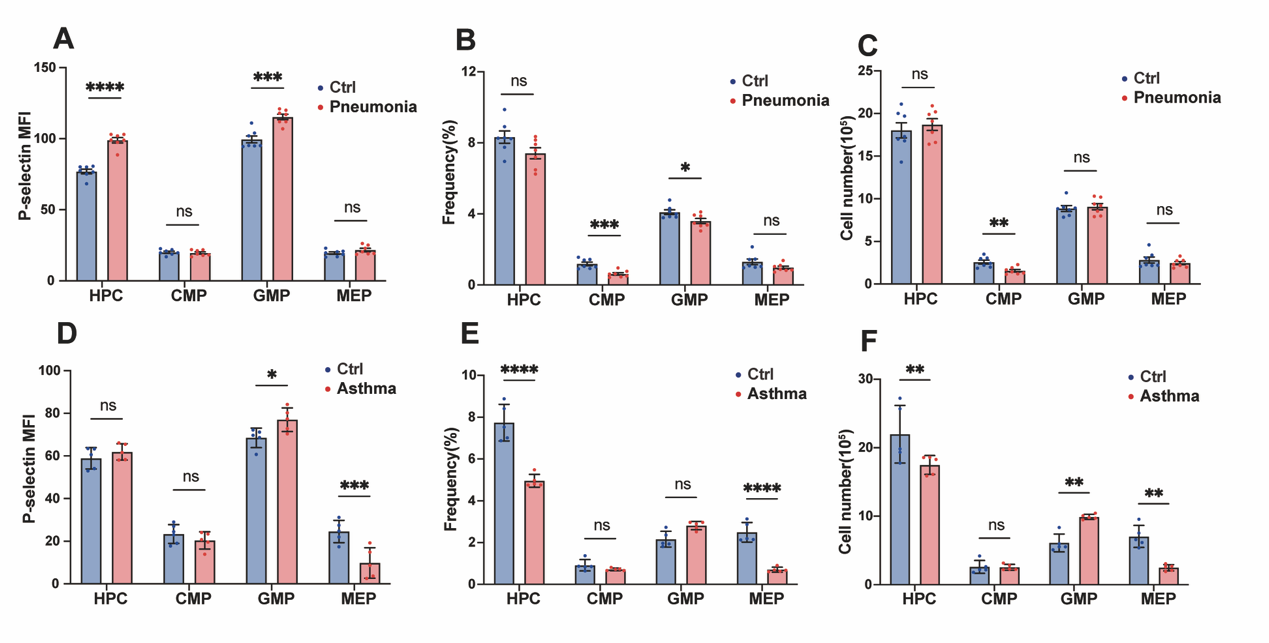
Supplementary Figure 1. The effect of inflammation on the hematopoietic system.** (**A**) P-selectin MFI in HPCs (CMPs, GMPs and MEPs) from pneumonia and Ctrl group (n=7 per group). (**B-C**) The percentage and number of HPCs (CMPs, GMPs and MEPs) pneumonia and Ctrl group (n=7 per group). (**D**) P-selectin MFI in HPCs (CMPs, GMPs and MEPs) from asthma and Ctrl group (n=5 per group). (**E-F**) The percentage and number of HPCs (CMPs, GMPs and MEPs) asthma and Ctrl group (n=5 per group). Error bars denote mean ± SEM. ns, no significance, **P* < 0.05, ***P* < 0.01, ****P* < 0.001, *****P* < 0.0001 (t-test).

**
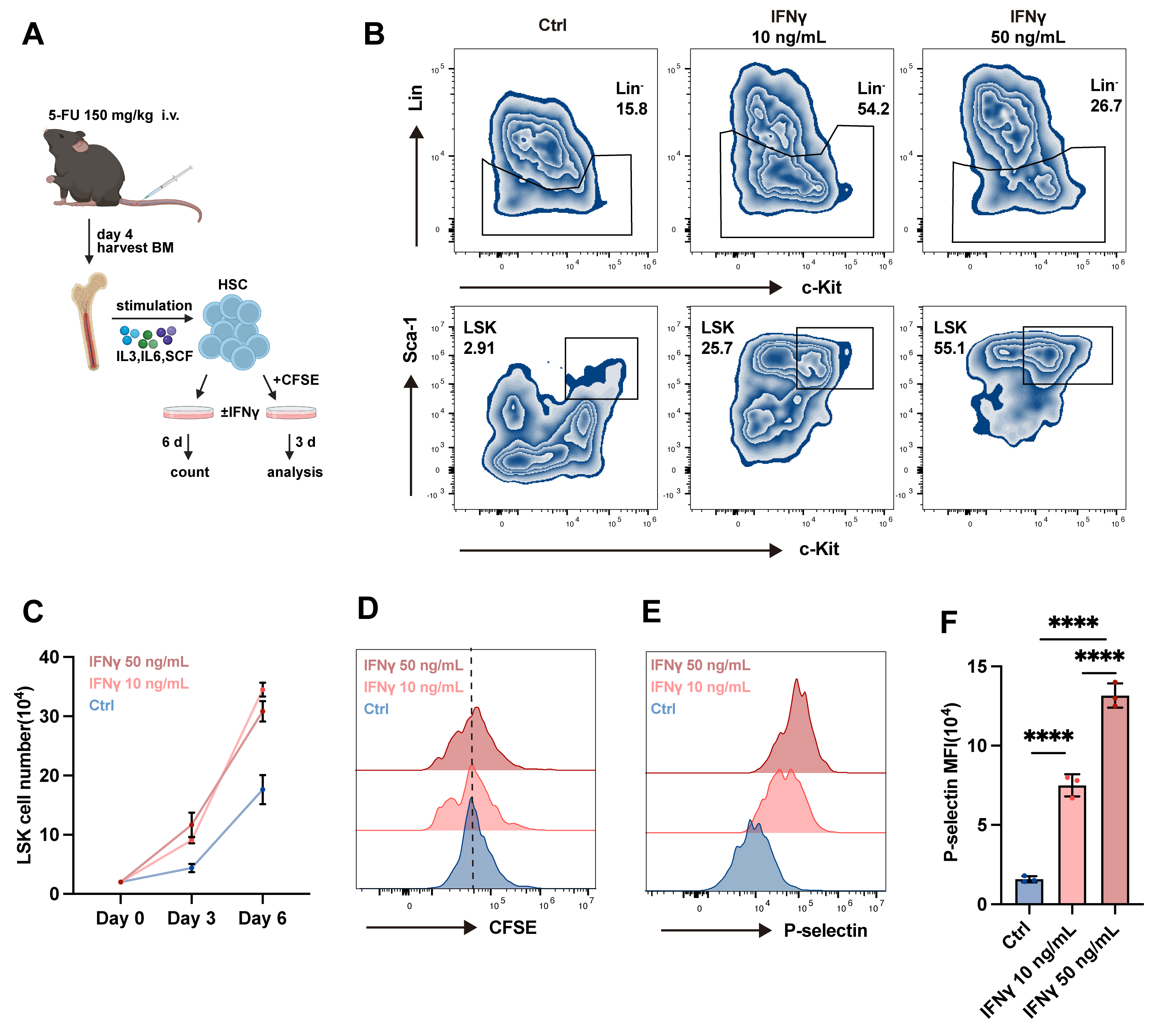
Supplementary Figure 2.** **INF-γ significantly promotes HSCs proliferation *in vitro*.** (A) Schematic overview of INF-γ treatment *in vitro*. (B) The percentage of HSC at day 6 in each group. (C) The number of HSC in each group (n=3 per group). (D) CFSE dilution assay after 3 days. (E) FACS analysis of P-selectin expression at day 6. (F) MFI analysis of P-selectin in each group at day 6 (n=3 per group). Error bars denote mean ± SEM, *****P* < 0.0001 (t-test).
